# Supplementary material for: The Construction and Exploration of a Comprehensive MicroRNA Centered Regulatory Network in Foxtail Millet (Setaria italica L.)
Source: Front Plant Sci. 2022 May 6;13:848474. doi: 10.3389/fpls.2022.848474 (PMC9121102; doi:10.3389/fpls.2022.848474)
Supplement: Supplementary file 2 [file Data_Sheet_2.zip › Sit-miR2275d_Seita.1G169800.1_351_TPlot.pdf]

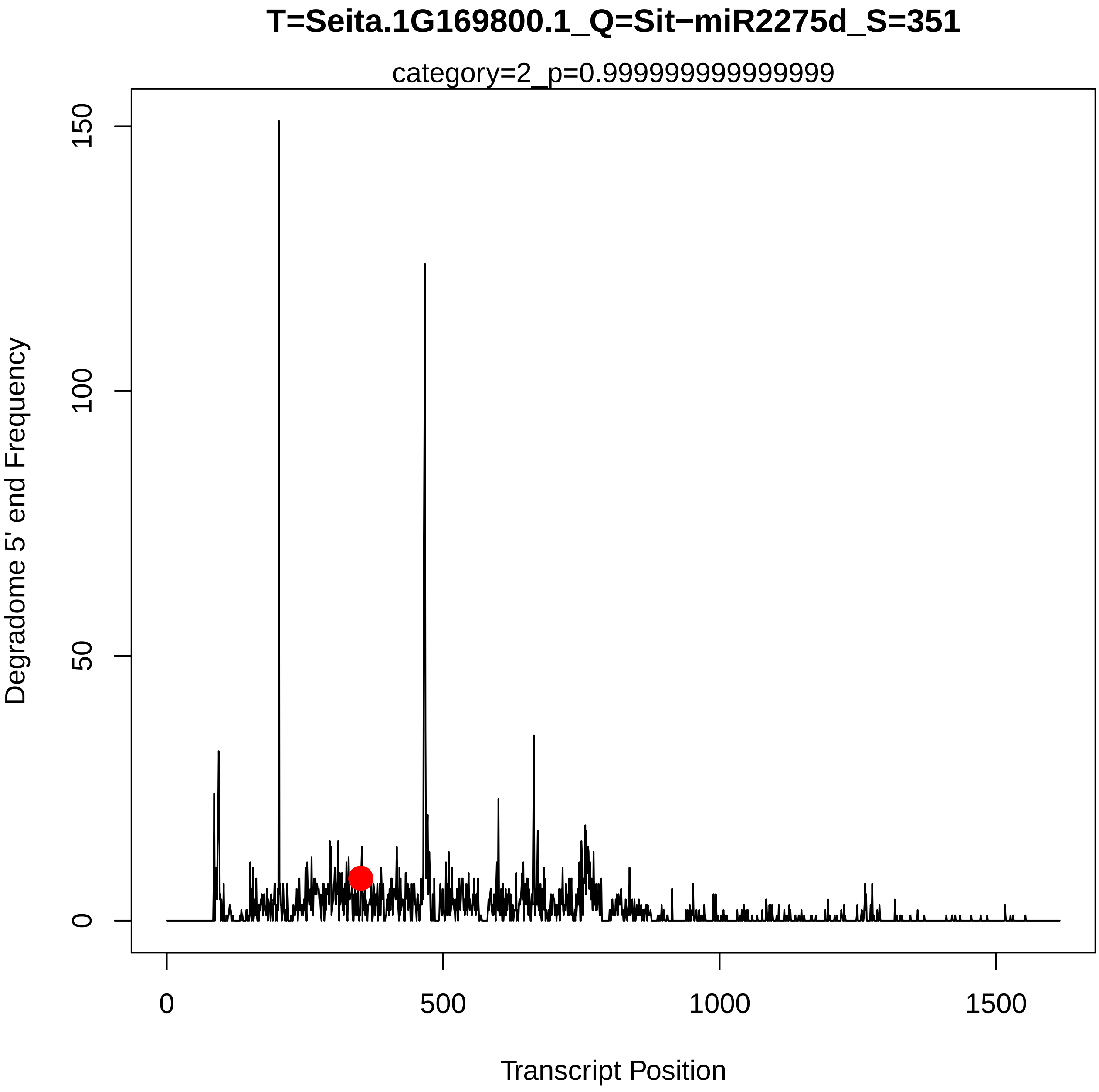
T=Seita.1G169800.1_Q=Sit−miR2275d_S=351
category=2_p=0.999999999999999
Degradome 5' end Frequency
0
50
100
150
0
500
1000
1500
Transcript Position
